# Cystosarcoma Phyllodes of the Seminal Vesicle: A Case Report and Literature Review

**DOI:** 10.1155/2014/302708

**Published:** 2014-01-02

**Authors:** Lucio Olivetti, Francesco Laffranchi, Vincenzo De Luca

**Affiliations:** ^1^Department of Radiology, Istituti Ospitalieri di Cremona, Viale Concordia 1, 26100 Cremona, Italy; ^2^Department of Radiology, University of Brescia, Spedali Civili, Brescia, Piazzale Spedali Civili 1, 25100 Brescia, Italy; ^3^Department of Urology, University of Brescia, Spedali Civili, Brescia, Piazzale Spedali Civili 1, 25100 Brescia, Italy

## Abstract

Cystosarcoma of the seminal vesicle is a very rare malignant tumor; in the literature only four cases are reported. We present a case of cystosarcoma phyllodes arising in the right seminal vesicle of a 49-year-old man without any urinary symptom but with persistent constipation. Ultrasound examination showed a mass at the right superior base of the prostate subsequently studied with CT and MRI. The patient underwent vesiculectomy; his postoperative course was uneventful. The patient is still well, without evidence of recurrent disease.

## 1. Introduction

The seminal vesicle is involved more frequently by tumors originating elsewhere, particularly prostatic carcinoma. Primary malignant seminal vesicle neoplasms are extremely uncommon: in addition to carcinomas and even more rare pure sarcomas, there is a distinctive group of mixed epithelial-stromal tumors that includes the cystosarcoma phyllodes reported in this case, exhaustively studied with ultrasound, computed tomography, and magnetic resonance.

## 2. Case Report

**Figure 1:**
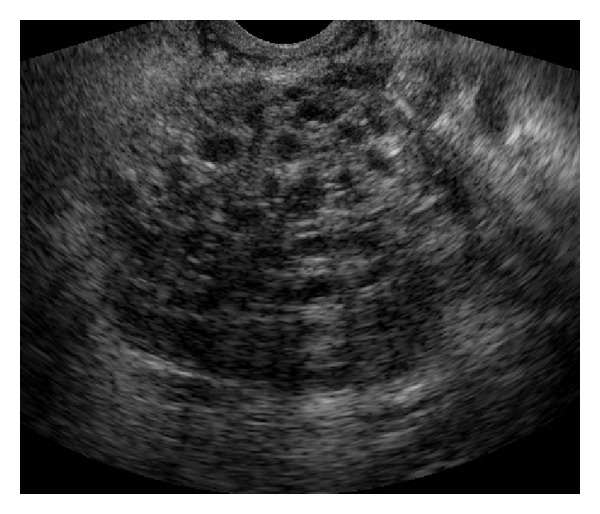
Transrectal ultrasound. Multiloculated solid mass of the right seminal vesicle.

A 49-year-old man was admitted with two months persistent constipation, incoercible with medical therapy. He did not report any urinary symptom, only a mild erectile dysfunction. On digital rectal examination his prostate was regular in size and consistency but with an extrinsic compression on the right-posterior wall by a mass. Transabdominal ultrasonography showed a localized cystic mass of about 7,3 × 5 cm, probably of the right seminal vesicle, characterized by multiple endoluminal septa. Transrectal US confirmed the origin of the lesion from the right seminal vesicle without infiltration of rectum, bladder, or prostate (Figure 1).

Routine blood exams, including PSA and prostatic acid phosphatase, were normal.

**Figure 2:**
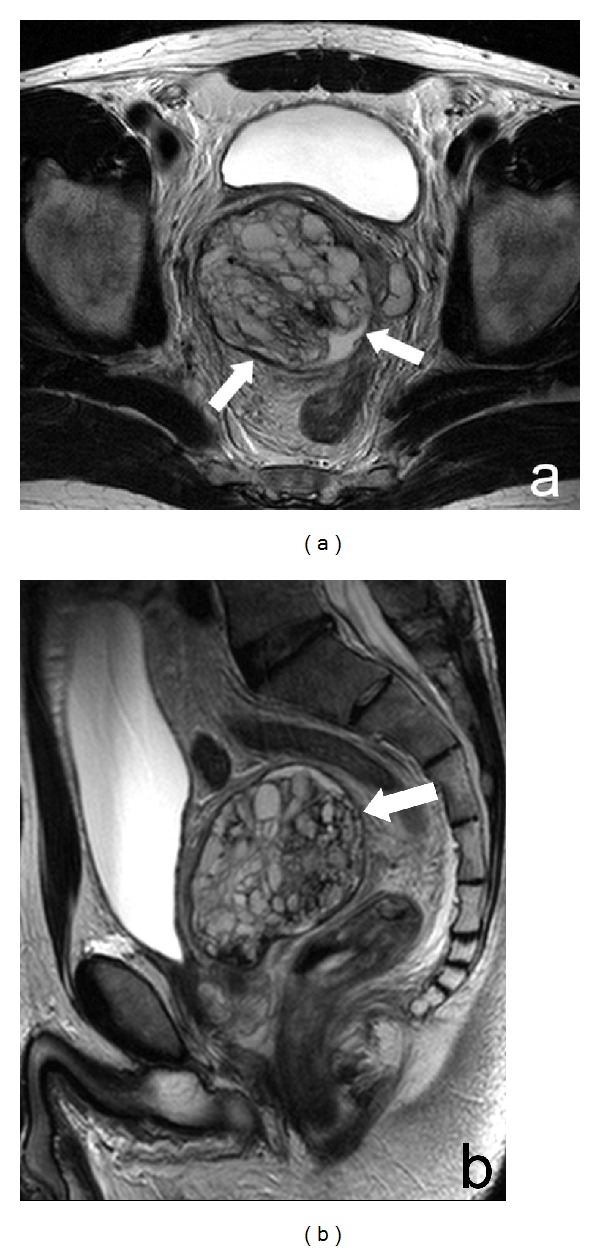
MRI of the pelvis. Axial (a) and sagittal (b) T2 sequence. Large mixed solid-fluid mass of the right seminal vesicle (arrows) without infiltration of adjacent structures.

Magnetic resonance (MR) was performed and reported a 56 × 63 × 59 mm lesion of the right seminal vesicle with a fluid content and endoluminal central solid tissue caracterized by a sepimentated structure with enhancement after Gadolinium-DTPA. On T1 weighted images, some hyperintensities in small cavities, caused probably by past hemorrhages, were observed. Prostate and bladder were normal, without signs of infiltration. No local adenopathies were found (Figure 2).

Colonoscopy confirmed a delayed fecal transit through the rectum and cystoscopy showed compression on the vesical neck without any alteration of the mucosa.

**Figure 3:**
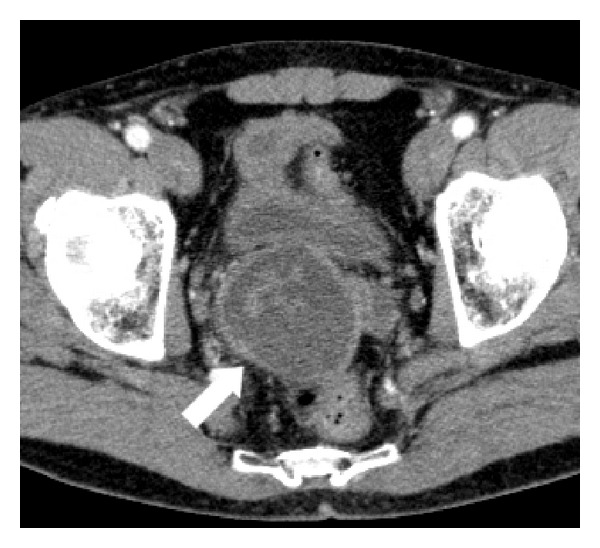
CT. Mixed mass of the right seminal vesicle (arrow).

Computed tomography (CT) staging was performed without any abnormalities of thoracic and abdominal organs; lesion of the right seminal vesicle was confirmed (Figure 3).

US guided needle biopsy was performed and it was negative for atypical cells.

The patient underwent right seminal vesiculectomy, with section of ipsilateral vas deferens and the saving of prostate, bladder, and rectum.

On gross examination the specimen consisted of a distorted and dilated seminal vesicle of 5 × 5 × 5 cm, with an intact and smooth external surface. On serial sectioning, in particular of the central mass of hard consistence, the lesion was characterized of necrotic-haemorragic areas with pseudocysts. Histologic examination demonstrated a biphasic tumor: one part with ischaemic necrosis, the other without necrosis characterized by an epithelial matrix mainly cystic delimited by addensed stroma (CD34+, vimentin +, actin ML +, estrogenic and progestinic receptors +) with atypic cells and a variable grade of mitosis. The proliferation index Ki 67 MIB1 clone was inhomogeneous with foci of high proliferation, in particular near the necrotic areas and associated with squamous metaplasia. The final histological diagnosis was cystosarcoma phyllodes of the right seminal vesicle.

Patient's recovery was uncomplicated, so after one week he was discharged.

Clinicians decided jointly for a strict clinical and CT follow up. Actually, after 14 months, the patient is still alive and makes his periodic clinical-strumental-laboratoristic controls without evidence of disease on chest and abdominal-pelvic CT.

## 3. Discussion

Reported primary malignant seminal vesicle neoplasms include a spectrum of carcinomas, sarcomas, and an uncommon group of tumors with mixed epithelial and stromal components.

Although rare, adenocarcinoma is the most common primary histotype. Because involvement of seminal vesicle by prostatic adenocarcinoma is a common occurrence (approximately in 12% of patients with clinically low stage prostate cancer on pathologic evaluation of their prostatectomy specimen), since 1956 Dalgaard and Giertsen applied strict criteria to diagnose primary neoplasms: the lesion must be a papillary or anaplastic carcinoma that is primarily localized in the seminal vesicle in absence of other primary carcinoma in the region (specifically, prostate or colorectal carcinoma) [1]. If a tumor large size prevents the accurate determination of the site of origin, seminal vesicle is distinguished from prostatic neoplasms by the absence of prostate specific antigen (PSA) staining [2].

Sarcomas are even rarer than those of epithelial origin. In the most conclusive documentation Schned et al. reviewed 11 cases of primary sarcoma of the seminal vesicle [3]. Many of the published cases are poorly documented due to the tendency of this neoplasm to widely invade neighboring structures, often obscuring the actual site of origin; it is possible that some may represent neoplasms arising from the pelvic soft tissue.

The third histotype is a rare group of tumors that contain both an epithelial and a stromal component and have been called epithelial-stromal or phyllodes tumors. Phyllodes tumors usually arise in the female breast and display a mixture of variably cellular stroma and benign glandular elements. The density and cytologic features of the stroma determine whether the tumor is a fibroadenoma, low-grade phyllodes tumor, or high-grade phyllodes tumor (cystosarcoma phyllodes). Phyllodes tumors with varying degrees of malignant potential have been reported in the prostate, including cystosarcoma phyllodes. Adenofibroma and adenosarcoma of the female genital tract are histologically similar to phyllodes tumors and are considered of müllerian origin [4].

In the seminal vesicle a range of epithelial-stromal neoplasms that is analogous to phyllodes tumors in the breast and prostate has been reported; they space from benign cystoadenoma and cystomyoma to cystosarcoma that is at the malignant end on the range of phyllodes tumors in the seminal vesicle.

In 1993 Fain et al. described the first case of cystosarcoma phyllodes of the seminal vesicle [4]. Since the submission of this paper, a similar case of müllerian adenosarcoma of the seminal vesicle was reported by Laurila et al. [5]. The review of the literature shows only few other cases as reported in Table 1.

Unlike previous cases, the patient subject of this report did not show any urogenital symptoms, and the only complaint was recently arisen constipation. Abdominal ultrasound first showed the existence of a pelvic mass with possible origin from the seminal vesicle, as confirmed by subsequent transrectal ultrasound. The lesion was also documented by CT (thorax, abdominal pelvis), however, as part of a staging to exclude metastases. Of course, MRI better explained the locoregional extension and hypothesized the possible phyllodes nature of the tumor. Like in Laurila's case and differently from Fain and Abe, the lesion was not solid but with a cystic component. On MRI the absence of infiltration of the adjacent structures obviously could not push the diagnosis up to define the high degree of malignancy and, therefore, the sarcomatous nature of the lesion. In our case, however, even the cytological examination did not show atypical cells, thus orienting the surgery to a simple vesiculectomy. Followup showed no recurrence, thus justifying the surgical choice that was made.

## Figures and Tables

**Table 1:** Reported cases of phyllodes tumor of moderate-high (cystosarcoma) grade.

Authors	Age. Symptoms	Radiological findings	Treatment	Gross findings	Followup
Fain et al. [4]	61. Acute urinary retention	US: solid mass replacing the left SV; CT: solid mass of high density	CPV	8 × 5 × 6.5 cm tan polypoid mass	Lung metastasis four years later

Laurila et al. [5]	49. Gradual decrease in urinary stream for several years	US: large fluid mass in the lower abdomen, initially misinterpreted as the urinary bladder; CT: cystic, fluid-filled mass located directly superior to the prostate and dorsal to the UB, replacing the right SV	CPV	6 × 5 × 5 cm cystic mass	NED four years later

Abe et al. [6]	65. Urinary hesitancy, frequency, and constipation	CT: solid mass involving nearly the entire right SV, compressing the prostate to the left anterior side, but distinct from it; EU: compression of the UB to the left anterior side	TV	5.5 × 6 solid mass (at CT)	Lung metastasis after seven months

Xu et al. [7]	59. Abdominal discomfort and symptoms of bladder outlet obstruction for 1 year	US: mass of the right SV; CT: mass with the density of soft tissue located posterior to the urinary bladder and anterior to the rectum; MR: soft-tissue, well-circumscribed mass	TV	9 × 8 × 6 cm capsulated mass with numerous cysts surrounded by solid areas with a glistening appearance	NED 22 months later

SV: seminal vesicle; UB: urinary bladder; CPV: cystoprostatoseminovesiculectomy; TV: tumorectomy and vesiculectomy; NED: no evidence of disease.
